# Epigenetic risk stratification in juvenile myelomonocytic leukemia by targeted methylation analysis of the *BMP4* locus

**DOI:** 10.1186/s13148-025-01983-0

**Published:** 2025-10-03

**Authors:** Foued Ghanjati, Annika Heck, Dirk Lebrecht, Peter Nöllke, Felicia Andresen, Natalia Rotari, Marlou Schoof, Maximilian Schönung, Daniel B. Lipka, Michael Dworzak, Barbara De Moerloose, Martina Sukova, Henrik Hasle, Kirsi Jahnukainen, Andrea Malone, Riccardo Masetti, Jochen Buechner, Marek Ussowicz, Albert Catala, Dominik Turkiewicz, Valérie de Haas, Markus Schmugge, Miriam Erlacher, Charlotte M. Niemeyer, Christian Flotho

**Affiliations:** 1 Department of Pediatric Hematology and Oncology, Children’s Hospital, Medical Center, Faculty of Medicine, University of Freiburg, Freiburg, Germany; 2 Section of Translational Cancer Epigenomics, Division of Translational Medical Oncology, German Cancer Research Center (DKFZ), Heidelberg, Germany; 3 National Center for Tumor Diseases (NCT), NCT Heidelberg, a partnership between, DKFZ and Heidelberg University Hospital, Heidelberg, Germany; 4 German Cancer Consortium (DKTK), DKFZ, Core Center, Heidelberg, Germany; 5 German Cancer Consortium (DKTK), Partner Site Freiburg, a partnership between, DKFZ and University Medical Center, Freiburg, Germany; 6 St. Anna Children’s Hospital, Department of Pediatrics and Adolescent Medicine, Anna Children’s Cancer Research Institute (CCRI), Medical University of Vienna, and St., Vienna, Austria; 7 Department of Paediatric Haematology-Oncology, Ghent University Hospital, Ghent, Belgium; 8 Department of Paediatric Haematology and Oncology, Second Faculty of Medicine, Charles University and University Hospital Motol, Prague, Czech Republic; 9 Department of Pediatrics, Aarhus University Hospital, Aarhus, Denmark; 10 Division of Hematology-Oncology and SCT Children´S Hospital, University of Helsinki and Helsinki University Hospital, Helsinki, Finland; 11 Pediatric Haematology, Our Lady’s Children’s Hospital, Dublin, Ireland; 12 Pediatric Oncology and Haematology, IRCCS Azienda Ospedaliero-Universitaria Di Bologna, Bologna, Italy; 13 Department of Pediatric Hematology and Oncology, Oslo University Hospital, Oslo, Norway; 14 Department of Pediatric Hematology and Oncology, BMT Unit CIC 817, Wroclaw Medical University, Wrocław, Poland; 15 Department of Hematology and Oncology, Hospital Sant Joan de Deu, Barcelona, Spain; 16 Department of Pediatrics, Section of Pediatric Oncology, Hematology, Immunology and Nephrology, Skåne University Hospital, Lund, Sweden; 17 Dutch Childhood Oncology Group, Princess Máxima Center for Pediatric Oncology, Utrecht, Netherlands; 18 Department of Hematology and Oncology, University Children’s Hospital, Zurich, Switzerland

**Keywords:** JMML, DNA methylation, *BMP4*, Bs-NGS, Risk stratification, Biomarker, Abstract word count = 217, Text word count = 3077

## Abstract

Juvenile myelomonocytic leukemia (JMML) is a rare pediatric myelodysplastic/myeloproliferative neoplasm characterized by distinct epigenetic signatures that facilitate molecular classification. This study aimed to evaluate the diagnostic utility of locus-specific DNA methylation in the bone morphogenetic protein 4 (*BMP4*) gene as a single predictor of disease outcomes in a cohort of 111 children diagnosed with JMML, alongside 9 healthy controls. Methylation levels of *BMP4*, assessed through targeted bisulfite next-generation sequencing (bs-NGS), were heterogeneous within the JMML cohort and were significantly associated with clinical risk factors, such as patient age, and fetal hemoglobin (HbF) levels. A comparative analysis of *BMP4* bs-NGS and genome-wide methylation array data revealed a strong positive correlation (*p* < 0.001). The sensitivity and specificity of *BMP4* bs-NGS for classifying high-methylation cases were 0.612 and 0.887, respectively. For *PTPN11*-mutant patients (*N *= 40), the sensitivity was 0.667 and the specificity was 0.842. Survival analysis indicated that patients with high *BMP4* methylation (*BMP4*h) had lower 5-year disease-free survival (DFS) rates than those with normal *BMP4* methylation (*BMP4*n). Specifically, the 20% of patients with highest *BMP4* methylation had a 5-year DFS of 0.38, in contrast to 0.62 for the lowest 20% (*p* = 0.007). These findings highlight the potential of *BMP4* methylation analysis as a complementary biomarker for JMML risk stratification, mirroring genome-wide methylation profiles known to associate with prognostic subgroups.

**Supplementary Information:**

The online version contains supplementary material available at 10.1186/s13148-025-01983-0.

## Background

JMML is a malignant clonal myelodysplastic/myeloproliferative disorder that manifests in infants and young children [1]. Allogeneic hematopoietic stem cell transplantation (HSCT) represents the sole curative therapy for the majority of JMML patients [2]. Previous studies conducted by the European Working Group of Myelodysplastic Syndromes in Childhood (EWOG-MDS) have demonstrated a 5-year event-free survival rate of 50–55% following HSCT, along with a 5-year cumulative incidence of relapse (CIR) of approximately 30–35% [3]. The molecular pathology of JMML involves early imbalance of RAS signal transduction, which is the central tumor-driving event [1]. This results in dysregulation of signaling pathways for growth and differentiation in multipotent hematopoietic stem/progenitor cells [4]. However, the initiating RAS pathway mutations do not fully capture JMML’s heterogeneity, as secondary genetic events, though infrequent, can contribute to disease complexity but are not always detectable [5–7]. Global DNA methylation analyses in JMML demonstrated a clear correlation between epigenetic alterations and both clinical outcome and RAS mutation subgroups [8–10]. By integrating the methylation levels of the 5,000 most variable CpG dinucleotides in JMML, a harmonized algorithm defined three distinct DNA methylation groups in JMML [11]. The low-methylation (LM) group is composed of patients with a favorable prognosis, including children with Noonan syndrome or a *CBL* mutation, as well as a subgroup of patients with an *NRAS* mutation. The intermediate-methylation (IM) group was found to be enriched for cases with a *KRAS* mutation and monosomy 7. Conversely, high methylation (HM) was associated with patients exhibiting an inferior prognosis, and often a *PTPN11* mutation. Our previous research into the role of specific cancer gene promoters using mass spectrometry has identified a clear correlation between DNA hypermethylation and poor clinical outcome [12]. The DNA methylation score developed by Lipka et al.[10] encompassed multiple CpG sites situated within the upstream sequence of the gene encoding bone morphogenetic protein 4 (*BMP4*), a signaling molecule within the transforming growth factor-beta (TGF-beta) superfamily [13, 14]. BMP4 plays a central role in early hematopoietic development, particularly in regulating hematopoietic stem cell proliferation and lineage commitment. Its epigenetic silencing has been implicated in various malignancies and may interact with dysregulated MAPK signaling, a hallmark of JMML. This biological relevance supports its investigation as a potential single-locus biomarker [15–17].

In the present study, we conducted a follow-up investigation utilizing targeted bisulfite next-generation sequencing (bs-NGS) to analyze leukemic cells from 111 patients with JMML or Noonan syndrome-associated myeloproliferative disorder (MPD) and nine young children without a hematologic malignancy for quantitative DNA methylation at *BMP4* as the single gene locus of interest. The data were compared to existing microarray-based genome-wide information on CpG methylation. The findings indicate that targeted *BMP4* methylation analysis by bs-NGS reflects JMML risk groups in a manner highly similar to genome-wide profiles.

## Methods

### Patients and samples

Samples were collected from 111 children diagnosed with JMML or Noonan syndrome-associated myeloproliferative disorder (MPD) according to the International Consensus Classification (ICC) [18, 19] and from nine children without hematological malignancy as controls. Consent was obtained from parents or legal guardians in accordance with the Declaration of Helsinki. Patients were participating in studies 98 and 2006 of the European Working Group of MDS in Childhood (EWOG-MDS; Clinical trial numbers: NCT00047268, registered on October 3, 2002, and NCT00662090, registered on April 17, 2008; www.clinicaltrials.gov; *N* = 101) or in the University of Freiburg "Hilda" pediatric biobank protocol. The studies were approved by institutional review committees at each contributing center.

To enable the comparison of *BMP4* methylation assessed by bs-NGS with the existing datasets of global methylation generated by Illumina array technology, we selected samples matching the patient series published previously [10]. Out of 147 patients in that cohort, identical DNA samples (i.e., aliquots from the same extraction) were available in 80 cases. DNA samples from 31 additional cases were used for concomitant *BMP4* bs-NGS and methylome arrays. Together, the 111-patient study cohort included 40 cases (36%) with somatic *PTPN11* mutation, 18 cases (16%) with somatic *KRAS mutation*, 19 cases (17%) with *NRAS* mutation, 13 cases (12%) with a clinical and molecular diagnosis of neurofibromatosis type 1 (NF1), 11 cases (10%) with Noonan syndrome/MPD and 10 cases (9%) with germline *CBL* mutation. Nine age-compatible (median age 5.3 years) bone marrow samples without hematological malignancy were included as controls. Global DNA methylation categories were determined according to the consensus definition described previously [11]. Based on array data, 31 cases (28%) were classified as HM, 35 cases (32%) as IM and 45 cases (40%) as LM. Additional details and patient characteristics are provided in Supplemental Table 1. Overall, the composition of the cohort reflected the typical clinical, molecular genetic and epigenetic landscape of JMML.

### Bisulfite conversion and target amplification

Bone marrow or peripheral blood samples were separated into mononuclear cells and granulocytes by density gradient centrifugation. Total genomic DNA (gDNA) was extracted from granulocytes using the QIAamp DNA Blood Mini Kit (Qiagen). 200 ng of gDNA were converted with sodium bisulfite and cleaned up using the EpiTect Plus DNA Bisulfite Kit (Qiagen). Primer oligonucleotides targeting the *BMP4* upstream region were designed using Primer3 and BLAST: 5’- GGTTGAGTATTTAGTTTGTTTTTT -3’ (*BMP4*_forward), 5’-TCACCATAAATCCCTACAATAAC- 3’ (*BMP4*_reverse). These primers are designed to amplify a region in the BMP4 gene located at chr14:53,958,034–53,958,169 in the human genome assembly hg38. PCR was performed using 40 ng of template bisulfite DNA and Platinum polymerase (Thermo Fisher) under the following thermal conditions: (1) Initial denaturation at 95 °C for 2 min, (2) denaturation at 95 °C for 30 s, (3) annealing at 56 °C for 30 s, (4) elongation at 72 °C for 30 s, 35 cycles (steps 2 to 4), (5) final extension at 72 °C for 10 min. Amplicons were then verified by 1.5% agarose gel electrophoresis.

### Bead purification

PCR products were purified using AMPure XP beads (Beckman Coulter). Product size assessment of a sample subset was performed by High Sensitivity DNA ScreenTape Analysis (Agilent) using the 4200 TapeStation (Agilent) and conventional 1.5% gel electrophoresis.

### Barcoding, size and quality assessment

Unique DNA barcode sequences and specific adapters for NGS were attached to each sample using the NEBNext Ultra II DNA Library Prep Kit for Illumina (New England Biolabs). Barcoded amplicons were purified using AMPure XP beads (Beckman Coulter). All samples were quantified using QuantiFluor ONE dsDNA dye (Promega) and a Quantus fluorometer (Promega).

### Library sequencing

All samples were combined into one library at equimolar ratios, which was then quantified using the QuantiFluor system (Promega). A diluted sample library, at 10 pM was denatured in 0.2 N NaOH and sequenced using the MiSeq v2 reagent kit (Illumina) at a read length of 2 × 250 bp with paired-end reads.

### Statistical analysis

FASTQ sequencing files underwent processing via CLC Genomics Workbench (Qiagen) for quality control, read trimming, mapping, and methylation quantification. Statistical analyses employed various methods to examine the data. Fisher's exact test assessed the relationship between *BMP4* bs-NGS and methylation array classes, while Kruskal–Wallis and Mann–Whitney tests evaluated continuous variables across groups and between two independent groups, respectively. Survival analyses, including disease-free survival (DFS), and cumulative incidence of relapse (CIR), utilized the Kaplan–Meier method in IBM SPSS Statistics (version 30). Results were expressed as 5-year probabilities with 95% confidence intervals. Multivariate analysis was conducted using the Cox proportional hazards regression model, including *BMP4* methylation status together with established prognostic factors (age at HSCT, somatic *PTPN11* mutation, and HbF level. Statistical analysis was performed using SPSS for Windows 30 (IBM) and NCSS 2004 (Number Cruncher Statistical Systems). Throughout the analyses, two-sided *p*-values below 0.05 were considered statistically significant. GraphPad Prism (version 9) generated graphs.

## Results

### *BMP4* gene methylation in JMML

**Fig. 1:**
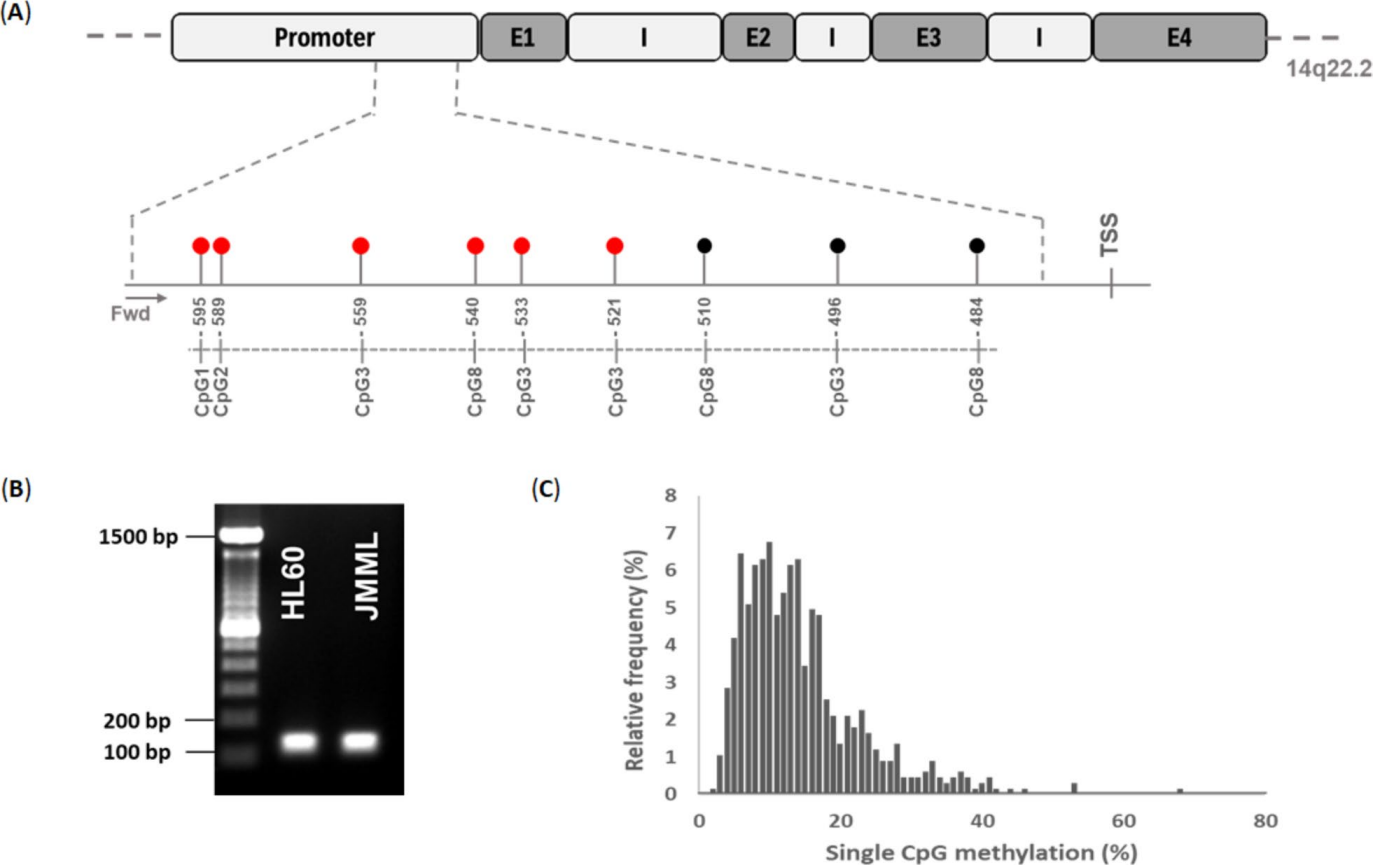
**A** Schematic illustration of the *BMP4* gene with promoter region, exons and introns along with the positions of nine CpG sites relative to the transcription start site (TSS). Arrows show the bisulfite-specific primers that amplify both methylated and unmethylated DNA. The first 6 CpGs (red) were variably methylated, the remaining 3 CpGs (black) were consistently unmethylated among JMML samples. **B** Gel electrophoresis (1.5% agarose) of *BMP4* PCR products from cell line HL60 and a JMML sample. **C** Frequency distribution of the methylation among all samples. The Y-axis shows the relative frequency while the x-axis reflects the individual methylation degree of the most variable 6 CpGs in the amplicon

**Fig. 2:**
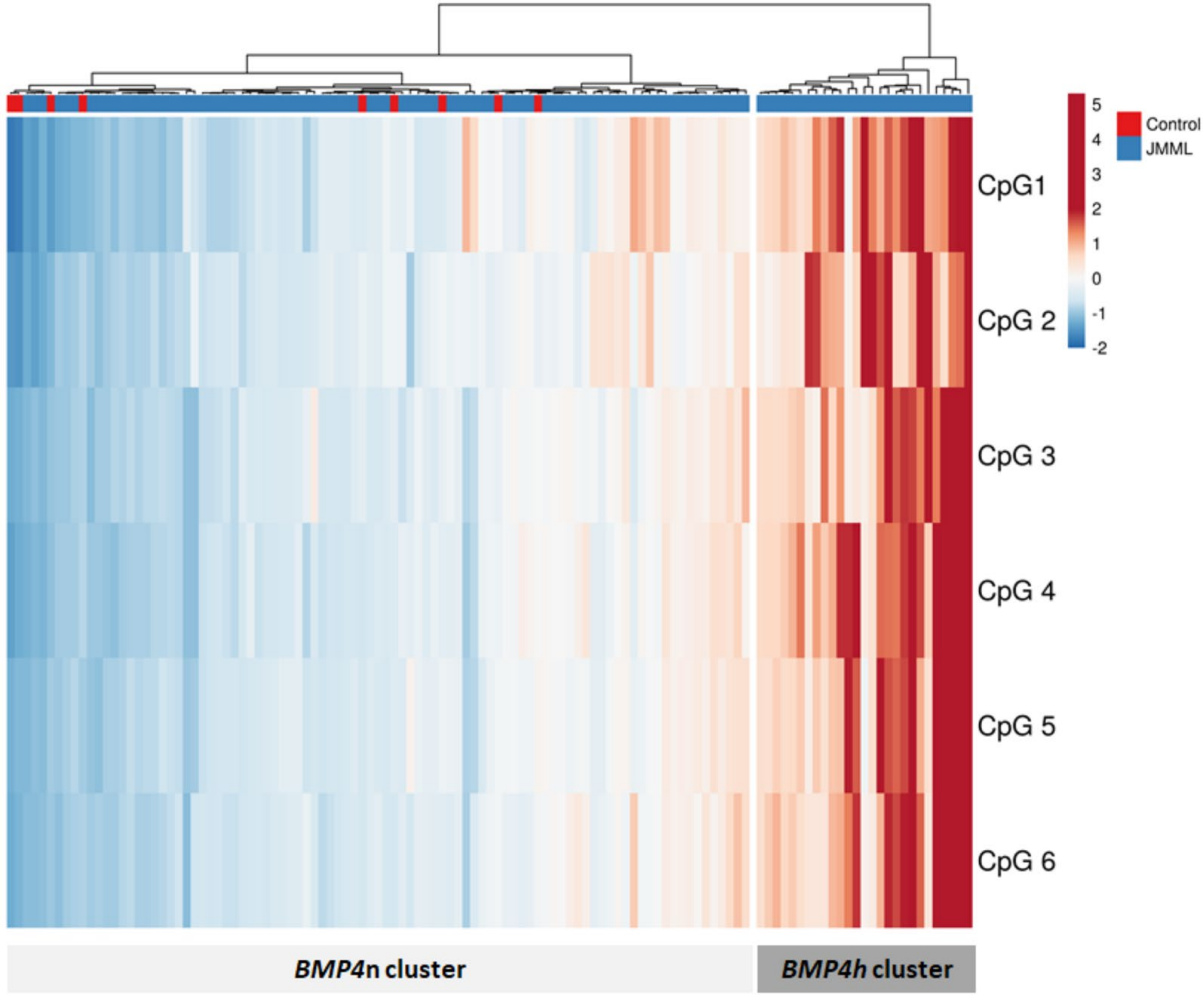
Unsupervised clustering of *BMP4* methylation as assessed by targeted bs-NGS. The heatmap comprises 120 columns, representing 111 JMML samples (blue) and 9 healthy controls (red), with rows corresponding to the 6 variable CpG sites. Column dendrograms were constructed using Manhattan distance and Ward linkage. The color scale indicates methylation intensity from low (blue) to high (red), with color-coded values corresponding to z-scores

**Fig. 3:**
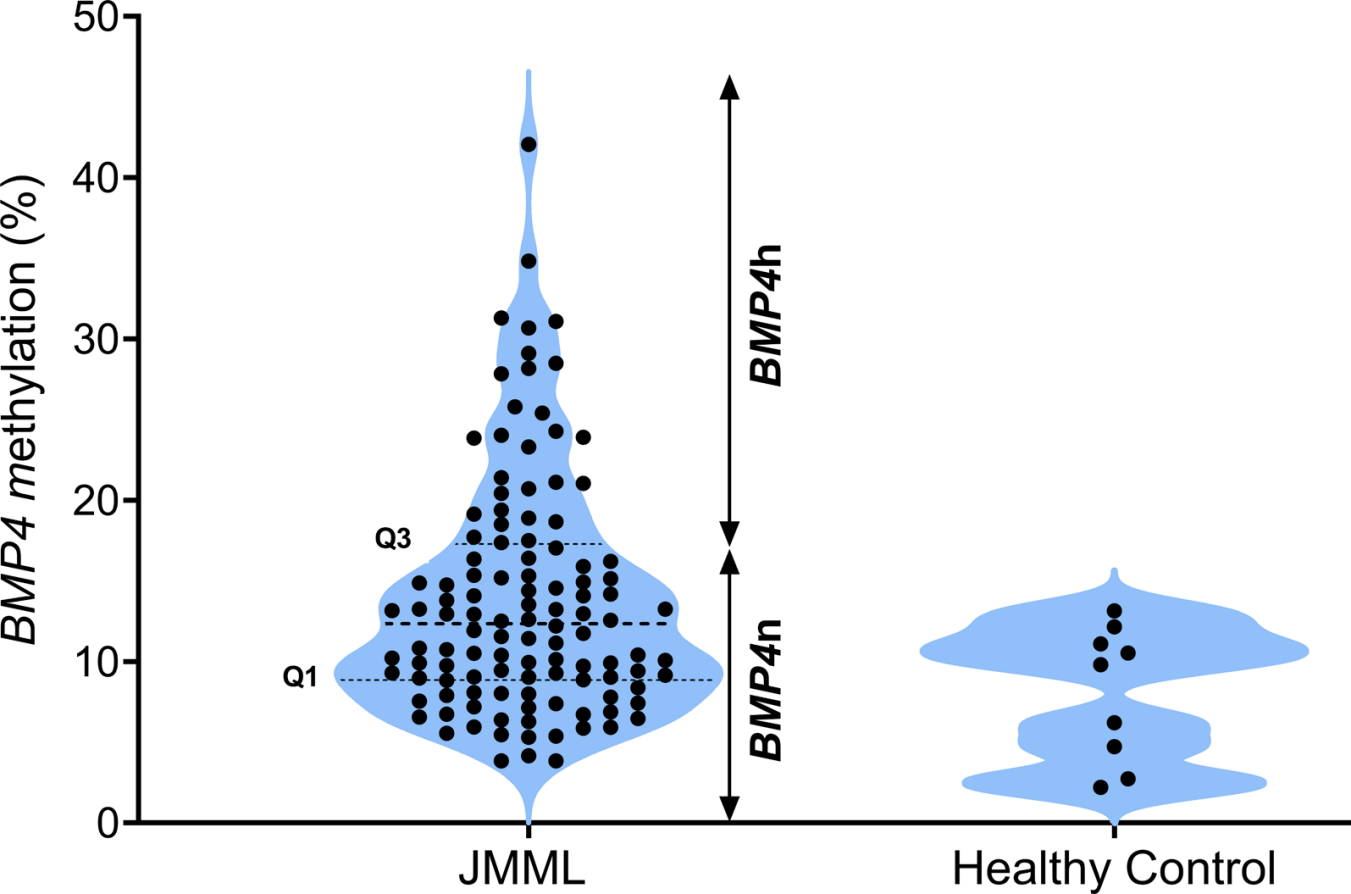
Violin plot of DNA methylation at the *BMP4* promoter region for JMML samples (*N* = 111) and healthy controls (*N* = 9) as assessed by targeted bs-NGS. The y-axis shows the average methylation value of 6 CpG dinucleotides and the width of each shape represents the density at that methylation level. Dashed lines indicate quartiles. Samples with methylation values higher than quartile 3 (Q3) were considered as high methylation (*BMP4*h). Methylation levels corresponding to quartiles 1 to 3 were considered normal (*BMP4*n)

Following thermodynamic and bioinformatic optimization of the bisulfite conversion and targeted amplification protocols (Fig. 1A, B), we performed *BMP4* bs-NGS on 111 retrospective samples. This approach generated data for 4,046,838 mapped CpG sites. The degree of cytosine methylation spanned a dynamic range of 4–42% (Fig. 1C**,** Figure S1). Unsupervised hierarchical clustering of the 111 JMML cases according to methylation levels at the most variable 6 CpG sites (Supplemental Table 2) revealed two major groups of samples, one with *BMP4* CpG sites exhibiting low-methylation levels (83 samples) and one with increased methylation (28 samples) (Fig. 2). Within the low-methylation group, a subordinate cluster formed that encompassed 25 samples with slightly higher *BMP4* methylation (Fig. 2, center). Although this picture appears to be consistent with the three-way methylome phenotype of JMML reported previously [9–11], the differences between the two *BMP4* low-methylation clusters were subtle and two of nine healthy control samples were assigned to the "low-to-intermediate" cluster, suggesting that single-locus *BMP4* methylation analysis has insufficient power to discriminate the intermediate-methylation JMML phenotype identified in genome-wide datasets [9–11]. Of note, one genome-wide study of JMML methylation by Japanese investigators also arrived at only two methylation groups [8]. We therefore forwent the definition of three *BMP4* methylation classes and combined the six CpGs into one methylation value per sample by arithmetic mean (Fig. 3). Using the nine control samples as a standard for normal *BMP4* methylation levels in hematopoietic cells and dividing the JMML data set into quartiles, we noted a broad overlap of JMML and controls. Consequently, we adopted a restrictive definition of high *BMP4* methylation and assigned quartiles 1–3 to the category of normal *BMP4* methylation (*BMP4*n) and quartile 4 to high *BMP4* methylation (*BMP4*h). Despite the different definitions, this proportion of *BMP4*h is compatible with that of the high-methylation group in the 292 patient genome-wide analysis published by Schönung et al. [11].

### *BMP4* methylation in JMML correlates with clinical, hematological, and genetic features

**Fig. 4:**
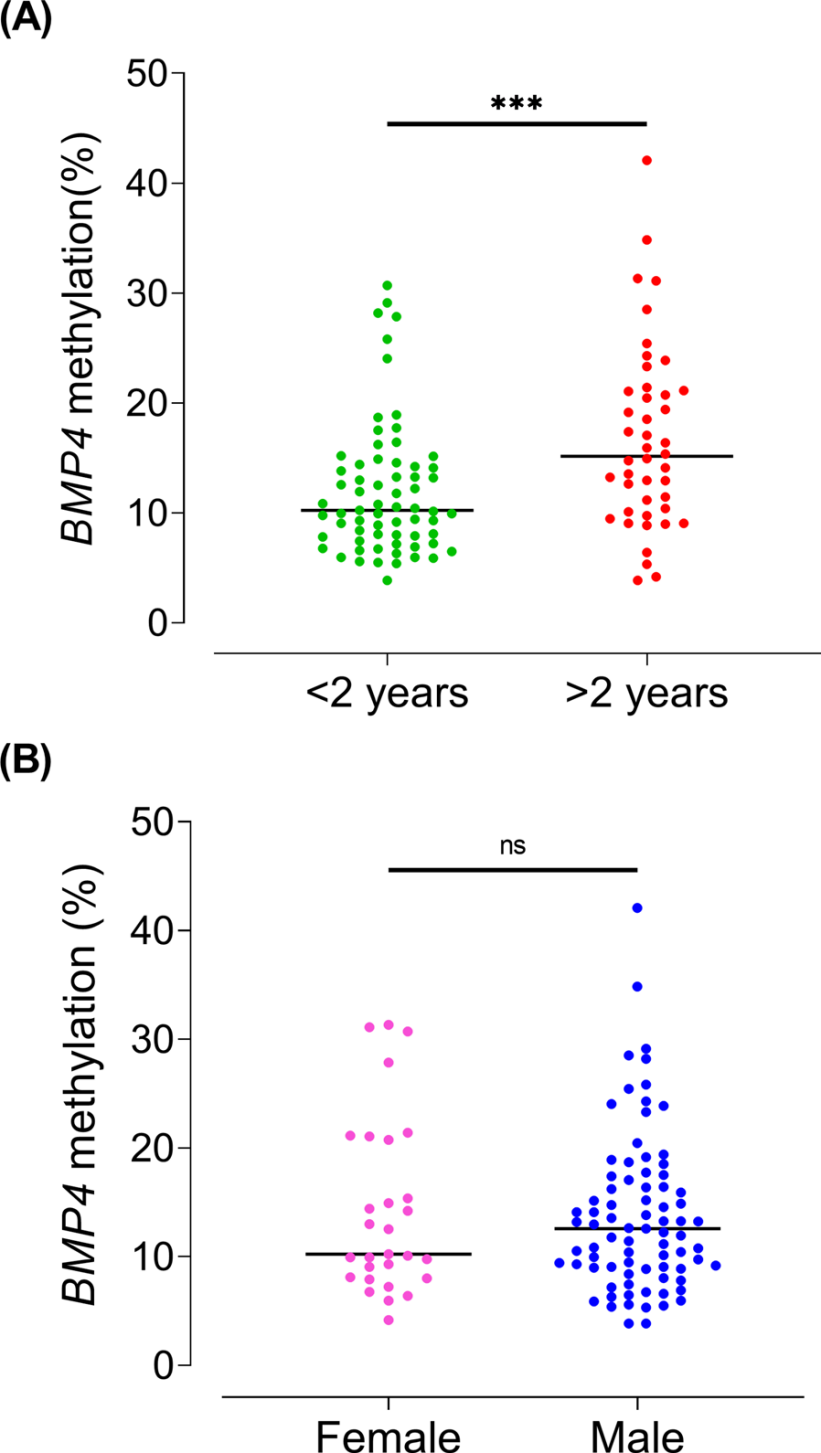
*BMP4* methylation levels of 111 JMML samples according to age (< 2 years and > 2 years) **A** and sex of the patients **B**, as assessed by targeted bs-NGS. Horizontal bars indicate median values. The significance of differences between groups was determined using the Mann Whitney test. Symbols: ***, *p* < 0.001; ns, not significant

**Fig. 5:**
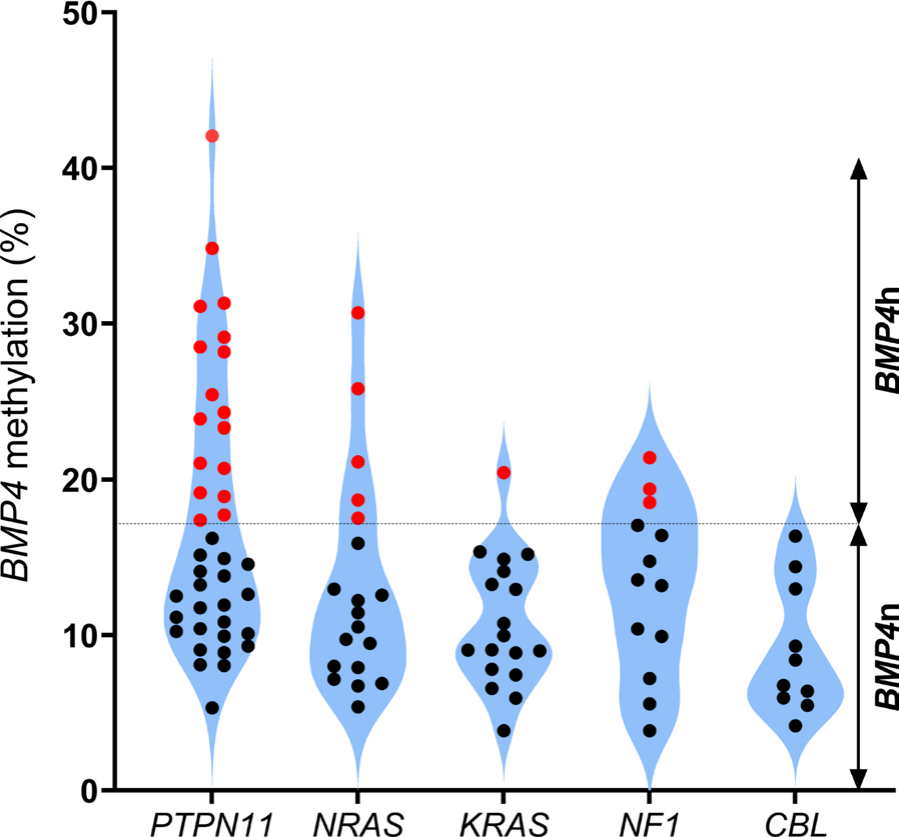
Proportion of *BMP4* methylation categories according to molecular subtypes of JMML

Next, we investigated the correlation of both categories *BMP4*n and *BMP4*h with clinical, hematological, and genetic features in patients with JMML. *BMP4* methylation levels correlated with age at diagnosis (Fig. 4A). Patients older than 2 years showed significantly higher *BMP4* methylation levels compared to those younger than 2 years (Mann–Whitney *p* < 0.05). In the *BMP*4h group, 64% of patients were older than 2 years (median age 2.7 years), while in the *BMP*4n group, 69% of patients were younger than 2 years (median age 1.1 years). This is concordant with the known association between global hypermethylation and age in JMML [11]. *BMP4* methylation did not differ between the two sexes (Fig. 4B). Additional hematological and genetic features associated with poor clinical outcome were more prevalent in the *BMP4*h group: This group of patients had lower platelet counts (median 55 G/l in *BMP4*h versus 96 G/l in *BMP4*n; Mann–Whitney *p* = 0.08) and presented higher levels of fetal hemoglobin [2] (median HbF: 28% [range, 0.5–80%] in *BMP4*h versus 8% [range, 0–68%] in *BMP4*n; Mann–Whitney *p* < 0.001). At the genetic level, subtypes associated with low-risk JMML [11] exhibited lower *BMP4* methylation (Fig. 5). Whereas all *CBL* cases were assigned to the *BMP4*n group, the *BMP4*h class was dominated by cases with somatic mutations in *PTPN11* (17 of 29 cases, 59%). Among 11 cases diagnosed with Noonan syndrome-associated MPD, two samples were classified as *BMP4*h.

### Comparison of *BMP4* methylation with global DNA methylation

**Fig. 6:**
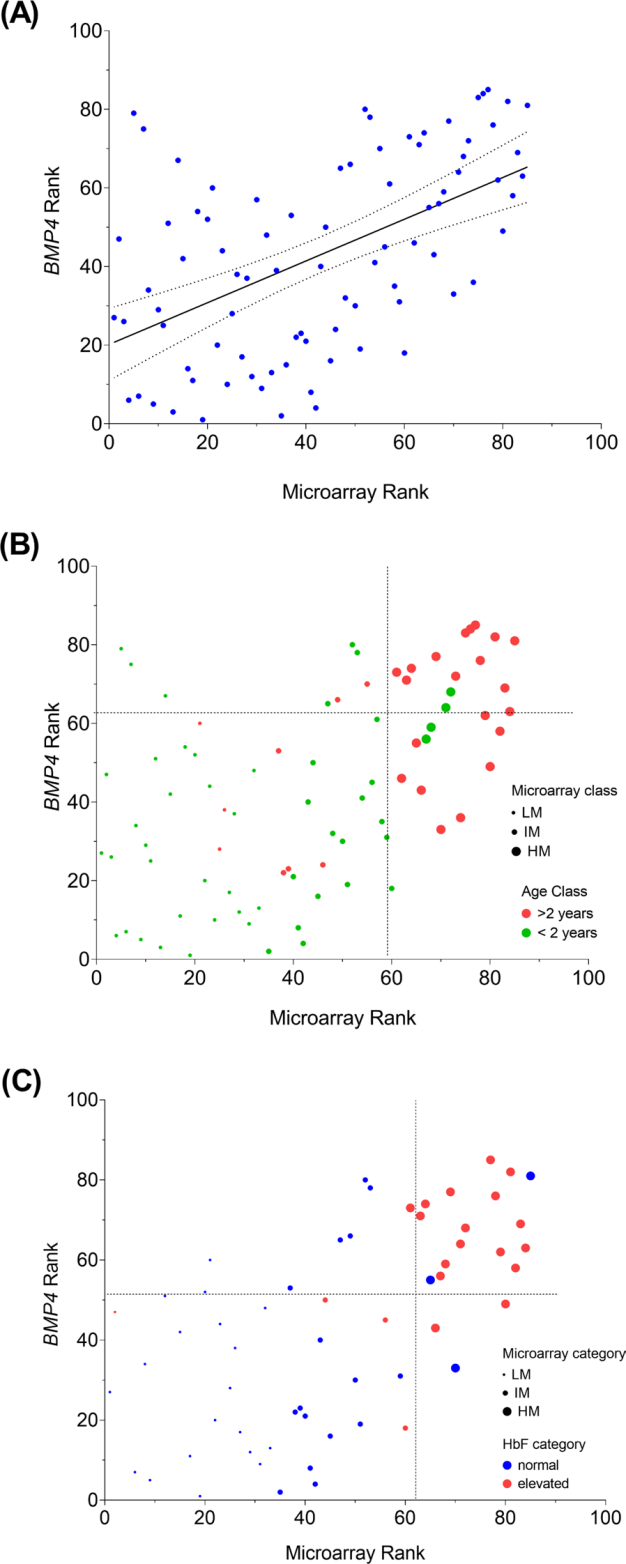
**A** Comparison of methylation rank orders determined by gene-specific *BMP4* bs-NGS or genome-wide microarray analysis in 111 JMML samples. Each dot represents an individual sample. **B** and **C** illustrate the association between microarray and *BMP4* bs-NGS methylation ranks in different age and HbF categories, respectively. The quadrants separate *BMP4*n / *BMP4*h and microarray non-HM / HM

In the next step of the analysis, we examined how well the methylation values determined by *BMP4* bs-NGS conformed with the methylome data from genome-wide methylation arrays. A direct comparison of numerical values was not meaningful due to different dynamic ranges of the two methods and the fact that the array data contained thousands of data points. We performed a cluster analysis of the methylomes as described in Schönung et al. [11] and rank-ordered the 111 samples according to position in the dendrogram. Likewise, the average methylation of 6 *BMP4* CpGs as measured by bs-NGS was rank-ordered across the 111 samples. The orders derived from both methods showed a strong positive correlation, *p* < 0.0001 (Fig. 6A). As expected, the high-methylation groups defined by either method were dominated by patients older than 2 years and with elevated levels of fetal hemoglobin at diagnosis (Fig. 6B, C). A Fisher exact test of independence was performed to examine the relation between methylation classes determined by *BMP4* bs-NGS and methylation array. This relation was highly significant (*p* < 0.001). Using the methylation array classification as the benchmark in the total cohort of 111 patients, *BMP4* bs-NGS demonstrated a sensitivity of 0.63 in correctly identifying high-methylation (HM) cases and a specificity of 0.89 in accurately classifying non-HM cases **(**Supplemental Table 3A). The corresponding positive predictive value (PPV) and negative predictive value (NPV) were 0.68 and 0.85, respectively (Supplemental Table 3A, Figure S2). In *PTPN11*-mutant cases (*N* = 40), the sensitivity was 0.67 and the specificity was 0.84, PPV 0.82, and NPV 0.70 (Supplemental Table 3B, Figure S2). There were one HM case and 17 non-HM cases in the *KRAS* group. All were classified correctly, so that sensitivity and specificity were calculated as 1.0, with corresponding PPV and NPV also being 1.0 (Supplemental Table 3C, Figure S2). In cases with *NRAS* mutation (*N* = 19), the numbers were sensitivity 0.6 and specificity 0.86, with PPV 0.60 and NPV 0.86 (Supplemental Table 3D, Figure S2) in NF1 cases (*N* = 13), sensitivity was 0.25 and specificity was 0.78, PPV 0.33, and NPV 0.7 (Supplemental Table 3E, Figure S2). All 10 patients with *CBL*-driven JMML were LM by array and *BMP4*n by bs-NGS (sensitivity, not applicable; specificity and NPV, 1.0). Using ≥ 15% as definition for elevated levels of HbF in patients older than 6 months [2], 44 patients had normal HbF whereas 24 patients presented with elevated HbF; in 43 cases, patients were younger than 6 months or HbF data were unavailable. In the group with normal HbF, 38 patients were non-HM by microarray and 34 of these were *BMP4*n (specificity, 0.9). Of the 24 patients with HbF ≥ 15%, 20 were array-HM cases and 14 were *BMP4*h (sensitivity, 0.7). We conclude that *BMP4* bs-NGS is overall a sufficient single predictor of "true" HM cases. However, in some HM cases, the genome-wide pattern is apparently not reflected in *BMP4* methylation, resulting in limited sensitivity of the locus-specific method. In addition, "false" *BMP4*n assignments inevitably occur in groups with an overrepresentation of HM cases due to the *BMP4*h definition as upper quartile.

### Association of *BMP4* methylation and clinical outcome

**Fig. 7:**
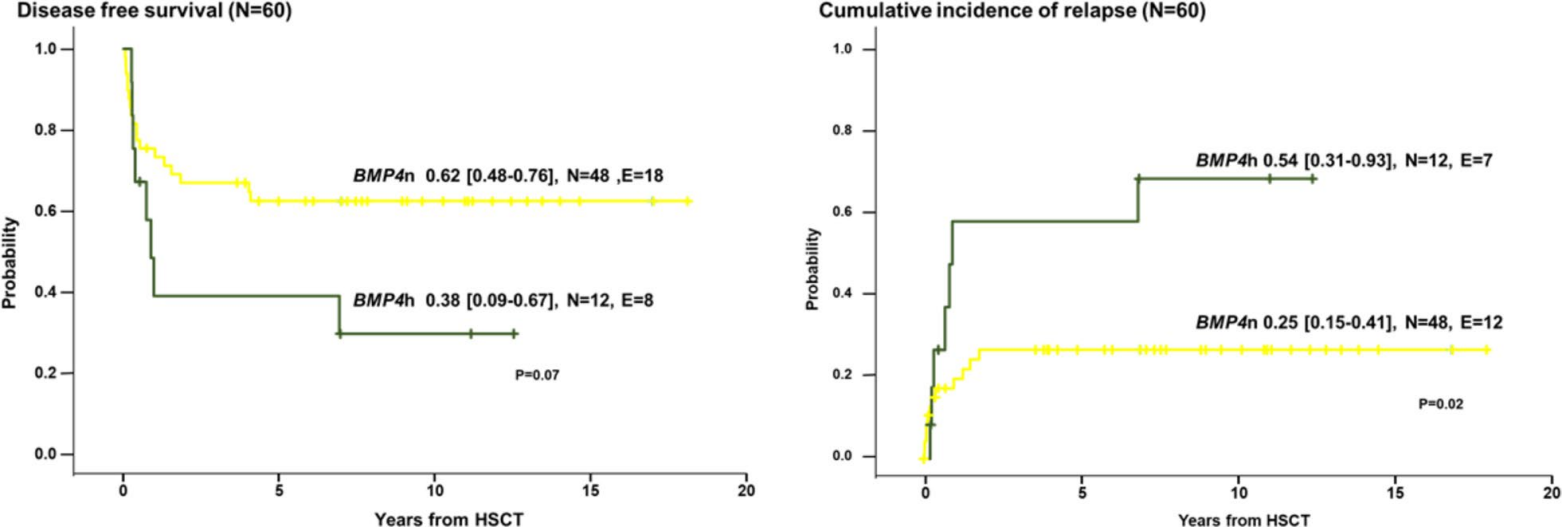
Kaplan–Meier curves showing the clinical outcome after HSCT of JMML patients according to *BMP4*h (green) and *BMP4*n (yellow) categories (*n* = 60). The numbers of individuals at risk (N) and the numbers of events (E) are indicated for each methylation category. Left, probability of DFS and right, CIR. Statistical significance was tested using log-rank (DFS) and Gray (CIR) tests

To perform survival analyses in patients following *BMP4* methylation, we selected 60 cases that had undergone allogeneic hematopoietic stem cell transplantation (HSCT). This cohort included 24 cases with somatic *PTPN11* mutation, 9 cases with somatic *KRAS* mutation, 11 cases with somatic *NRAS* mutation, 10 cases with NF1 and 6 cases with *CBL* mutation*.* The median time of follow-up since HSCT was 7.5 years (range, 0.5–18.7 years). Patients who were alive and disease-free were censored at the time of last follow-up. Other factors known to influence the risk of relapse such as the stem cell donor and the type of preparative procedure were not considered during case selection. The probability of 5-year disease-free survival in the total group of 60 patients was 57% (95%-CI, 44%–70%). We observed a significant difference in outcome depending on *BMP4* methylation status: The 5-year disease-free survival was 62% (95%-CI, 48%–76%) in the *BMP4*n group (*N* = 48) whereas it was only 38% (95% CI, 9%–67%) in the *BMP*4h group (*N* = 12) (log-rank test *p* = 0.07). Given the observed association of *BMP4* methylation with known prognostic factors for relapse, primarily HbF level and *PTPN11*, we tested whether the *BMP4* methylation might also reflect the risk of relapse after HSCT. As expected, the 20% of patients with the highest *BMP4* methylation showed a significantly higher 5-year incidence of relapse (54%; 95%-CI, 31%–93%) compared with all other patients (25%; 95%-CI, 15%–41%) (*p* = 0.02) (Fig. 7). Multivariable Cox regression including age at HSCT, *PTPN11* mutation status, HbF level, and *BMP4* methylation status did not identify any factor as independently significant. *PTPN11* mutation showed the strongest effect (relative risk [RR] 2.2, 95% CI 0.9–5.0, *p* = 0.07). *BMP4* high methylation carried a relative risk of 1.4 (95% CI 0.5–4.0, not significant [n.s.]), similar to HbF (RR 1.3, 95% CI 0.5–3.6, n.s.) or age at HSCT (RR 1.0, 95% CI 0.4–2.4, n.s.).

## Discussion

### Correlation of *BMP4* methylation with clinical risk factors

The frequent occurrence of *BMP4* hypermethylation in various hematological malignancies makes it a promising candidate as biomarker for diagnosis, prognosis, and monitoring treatment [20]. Previous studies identified *BMP4*, *CALCA*, *CDKN2B*, and *RARB* as frequently hypermethylated genes in JMML, with *BMP4* showing aberrant methylation in approximately one-third of patients [12, 21]. The current study aimed to evaluate *BMP4* methylation as a single predictor in JMML using a well-documented cohort, employing targeted bs-NGS. We analyzed aberrant DNA methylation at the *BMP4* promoter in leukemic cells from 111 JMML patients and nine children without hematological malignancy as controls, building on previous investigations which had demonstrated a robust correlation between clinical parameters indicative of poor prognosis in JMML and global DNA hypermethylation across numerous CpG sites [8–10, 12]. Overall, the degree of *BMP4* promoter CpG methylation was low in our study, with only a small proportion of samples exhibiting high-methylation levels. Elevated *BMP4* methylation correlated with patient age and fetal hemoglobin (HbF) levels, which are both established predictors of reduced survival, highlighting *BMP4* as a biomarker for clinical risk in JMML.

By multivariable Cox regression, *BMP4* methylation did not reach independent significance, which is not unexpected given the limited cohort size and the dominance of *PTPN11* mutations as a prognostic factor.

### *BMP4* methylation reflects genome-wide methylation changes in JMML

Although one would not expect the methylation status of 6 CpG positions within the *BMP4* promoter region to fully represent the entire variability of all ~ 5000 JMML-specific CpGs defined previously [10, 11], the comparison between focal and global approaches to methylation analysis yielded interesting results. The cluster analysis and rank-ordering of 111 samples demonstrated a clear positive correlation between *BMP4* locus-specific and global methylation patterns, particularly evident in patients with *PTPN11* and *CBL* subtypes and those with elevated HbF levels. The *BMP4* bs-NGS assay showed promising predictive capabilities, with an overall specificity of 0.89 and a sensitivity of 0.63 in classifying cases with high genome-wide methylation level when benchmarked against the array-based data. While the high specificity of *BMP4* methylation supports its diagnostic robustness, the moderate sensitivity limits its utility as a standalone prognostic biomarker. Therefore, we consider *BMP4* methylation most valuable as a complementary marker when integrated with clinical and molecular features. However, the performance varied across different genetic subgroups. Particularly, samples with *PTPN11* mutations and elevated HbF which also had high *BMP4* methylation were strongly associated with global hypermethylation. Conversely, low *BMP4* methylation was predominantly linked to low global methylation, especially in cases with *CBL* mutations. While genome-wide methylation profiling typically requires 3–4 weeks and substantial resources, targeted *BMP4* methylation analysis can be completed within 48 h in standard molecular biology facilities and can be run in parallel with the targeted NGS procedures carried out to determine the genetic JMML subtype.

### *BMP4* methylation versus comprehensive profiling

JMML risk stratification has bridged the gap between clinical and molecular assessment in recent years, largely due to the advent of global DNA methylation profiling. Several approaches to categorization of DNA methylation in JMML were reported, such as the machine learning-based classifier by Kitazawa et al. [22], the MethylSeq classifier comprising 3,000 CpG sites [11], and our own array-based classifier [10, 11]. However, applying these comprehensive techniques in resource-limited laboratories poses practical challenges. Our results suggest that *BMP4* methylation as a single parameter holds the promise to offer a simple alternative. Despite its potential, however, single-locus analysis of *BMP4* methylation showed limitations in recognizing the intermediate-methylation group described previously [10]. This issue was also encountered by others; for example, the group developing the Digital Restriction Enzyme Analysis of Methylation (DREAM) method reported difficulties in reliably identifying the IM group [22]. The subtle dissimilarities within low-methylation clusters and misclassification of some array-HM cases into the *BMP4* "low-to-intermediate" cluster confirm the expected limitations of streamlined approaches focused on a single gene or a set of few genes. That said, the notable correlation between *BMP4* methylation status and genome-wide methylation array data still suggests that focal methylation at the *BMP4* locus may reflect relevant epigenetic processes in JMML. We also acknowledge that the genetic homogeneity of our largely European ancestry cohort may limit the generalizability of quartile-based cutoff values to more diverse populations, and emphasize the need for external validation before clinical application of this thresholding approach.

## Conclusions

The findings presented here suggest that locus-specific aberrant DNA methylation, as exemplified for the *BMP4* promoter, correlates to a large extent with broader genome-wide methylation patterns in JMML and may thus aid in diagnosing high- or low-risk JMML. Prospective studies are required to determine the optimal threshold for *BMP4* methylation to be used for risk assessment.

## Supplementary Information

Additional file 1.Frequency distribution of the methylation according the genetic subtype. The Y-axis shows the relative frequency while the x-axis reflects the individual methylation degree of the most variable 6 CpGs in the amplicon.

Additional file 2. Bar graphs representing the performance metrics of *BMP4* bs-NGS in classifying methylation status for each genetic subtype of JMML. The y-axis shows the values for sensitivity, specificity, PPV, and NPV, while the x-axis represents the four genetic subtypes (PTPN11, KRAS, NRAS, and NF1). Each subtype has four bars, corresponding to the four performance metrics.

Additional file 3.

Additional file 4.

Additional file 5.

Additional file 6.

## Data Availability

All data generated or analyzed during this study are included in this published article and its supplementary information files.

## Abbreviations:

JMML: Juvenile myelomonocytic leukemia
MPD: Noonan syndrome-associated myeloproliferative disorder
BMP4: Bone morphogenetic protein 4
bs-NGS: Bisulfite next-generation sequencing
HbF: Fetal hemoglobin
HSCT: Hematopoietic stem cell transplantation
EWOG-MDS: European Working Group of Myelodysplastic Syndromes Childhood
LM: Low methylation
IM: Intermediate methylation
HM: High methylation
TGF-beta: Transforming growth factor-beta
gDNA: Genomic DNA
*BMP4*n: *Normal BMP4* methylation
*BMP4*h: High *BMP4* methylation

## References

[1] Niemeyer CM, Flotho C. Juvenile myelomonocytic leukemia: who’s the driver at the wheel? Blood. 2019;133(10):1060–70. 30670449 10.1182/blood-2018-11-844688

[2] Niemeyer, C.M., et al. 1997 *Chronic myelomonocytic leukemia in childhood: a retrospective analysis of 110 cases. European Working Group on Myelodysplastic Syndromes in Childhood (EWOG-MDS).* Blood. 89(10) 3534–43. 9160658

[3] Locatelli F, et al. Hematopoietic stem cell transplantation (HSCT) in children with juvenile myelomonocytic leukemia (JMML): results of the EWOG MDS/EBMT trial. Blood. 2005;105(1):410–9. 15353481 10.1182/blood-2004-05-1944

[4] Flotho C, et al. RAS mutations and clonality analysis in children with juvenile myelomonocytic leukemia (JMML). Leukemia. 1999;13(1):32–7. 10049057 10.1038/sj.leu.2401240

[5] Sakaguchi H, et al. Exome sequencing identifies secondary mutations of SETBP1 and JAK3 in juvenile myelomonocytic leukemia. Nat Genet. 2013;45(8):937–41. 23832011 10.1038/ng.2698

[6] Hofmans M, et al. The long non-coding RNA landscape in juvenile myelomonocytic leukemia. Haematologica. 2018;103(11):e501–4. 29858388 10.3324/haematol.2018.189977 PMC6278979

[7] Caye A, et al. Juvenile myelomonocytic leukemia displays mutations in components of the RAS pathway and the PRC2 network. Nat Genet. 2015;47(11):1334–40. 26457648 10.1038/ng.3420

[8] Murakami N, et al. Integrated molecular profiling of juvenile myelomonocytic leukemia. Blood. 2018;131(14):1576–86. 29437595 10.1182/blood-2017-07-798157

[9] Stieglitz E, et al. Genome-wide DNA methylation is predictive of outcome in juvenile myelomonocytic leukemia. Nat Commun. 2017;8(1):2127. 29259179 10.1038/s41467-017-02178-9 PMC5736624

[10] Lipka DB, et al. RAS-pathway mutation patterns define epigenetic subclasses in juvenile myelomonocytic leukemia. Nat Commun. 2017;8(1):2126. 29259247 10.1038/s41467-017-02177-w PMC5736667

[11] Schonung M, et al. International consensus definition of DNA methylation subgroups in juvenile myelomonocytic leukemia. Clin Cancer Res. 2021;27(1):158–68. 33139265 10.1158/1078-0432.CCR-20-3184 PMC7785676

[12] Olk-Batz C, et al. Aberrant DNA methylation characterizes juvenile myelomonocytic leukemia with poor outcome. Blood. 2011;117(18):4871–80. 21406719 10.1182/blood-2010-08-298968

[13] Chang H, Brown CW, Matzuk MM. Genetic analysis of the mammalian transforming growth factor-beta superfamily. Endocr Rev. 2002;23(6):787–823. 12466190 10.1210/er.2002-0003

[14] Kawabata M, Imamura T, Miyazono K. Signal transduction by bone morphogenetic proteins. Cytokine Growth Factor Rev. 1998;9(1):49–61. 9720756 10.1016/s1359-6101(97)00036-1

[15] Sadlon TJ, Lewis ID, D’Andrea RJ. BMP4: its role in development of the hematopoietic system and potential as a hematopoietic growth factor. Stem Cells. 2004;22(4):457–74. 15277693 10.1634/stemcells.22-4-457

[16] Goldman DC, et al. BMP4 regulates the hematopoietic stem cell niche. Blood. 2009;114(20):4393–401. 19759357 10.1182/blood-2009-02-206433 PMC2777124

[17] Jeong S, et al. BMP4 and perivascular cells promote hematopoietic differentiation of human pluripotent stem cells in a differentiation stage-specific manner. Exp Mol Med. 2020;52(1):56–65. 31956269 10.1038/s12276-019-0357-5 PMC7000736

[18] Mayerhofer C, Niemeyer CM, Flotho C. Current treatment of juvenile myelomonocytic leukemia. J Clin Med. 2021. 10.3390/jcm10143084. 34300250 10.3390/jcm10143084 PMC8305558

[19] Arber DA, et al. International consensus classification of myeloid neoplasms and acute leukemias: integrating morphologic, clinical and genomic data. Blood. 2022;140(11):1200–28. 35767897 10.1182/blood.2022015850 PMC9479031

[20] Ordway JM, Williams K, Curran T. Transcription repression in oncogenic transformation: common targets of epigenetic repression in cells transformed by Fos Ras or Dnmt1. Oncogene. 2004;23(21):3737–48. 14990994 10.1038/sj.onc.1207483

[21] Sakaguchi H, et al. Aberrant DNA methylation is associated with a poor outcome in juvenile myelomonocytic leukemia. PLoS ONE. 2015;10(12):e0145394. 26720758 10.1371/journal.pone.0145394 PMC4697810

[22] Kitazawa H, et al. Simple and robust methylation test for risk stratification of patients with juvenile myelomonocytic leukemia. Blood Adv. 2021;5(24):5507–18. 34580726 10.1182/bloodadvances.2021005080 PMC8714717

