# Supplementary figures and images for: Epigenetic risk stratification in juvenile myelomonocytic leukemia by targeted methylation analysis of the BMP4 locus
Source: Clin Epigenetics. 2025 Oct 3;17:154. doi: 10.1186/s13148-025-01983-0 (PMC12492826; doi:10.1186/s13148-025-01983-0)

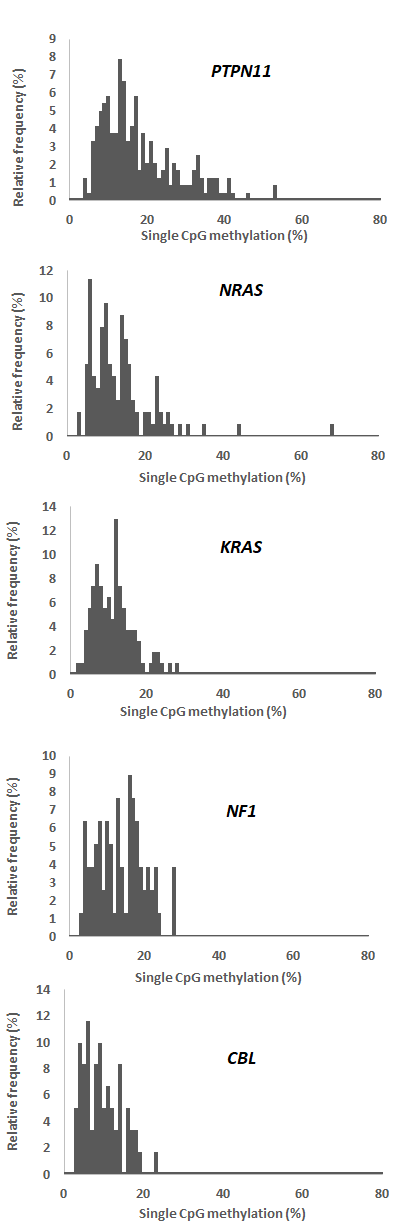

Supplement: Supplementary file 1 — Additional file 1.Frequency distribution of the methylation according the genetic subtype. The Y-axis shows the relative frequency while the x-axis reflects the individual methylation degree of the most variable 6 CpGs in the amplicon. [file 13148_2025_1983_MOESM1_ESM.tif]

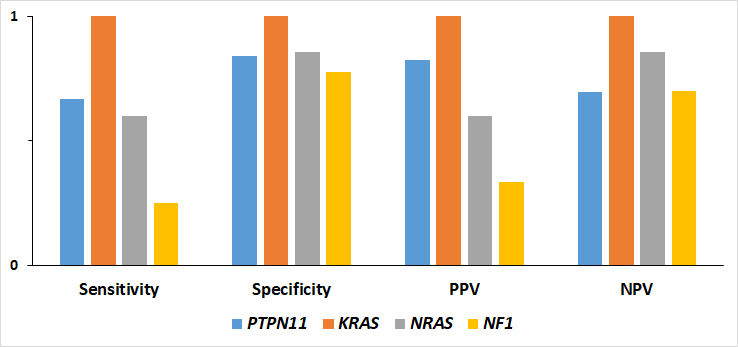

Supplement: Supplementary file 2 — Additional file 2. Bar graphs representing the performance metrics of BMP4 bs-NGS in classifying methylation status for each genetic subtype of JMML. The y-axis shows the values for sensitivity, specificity, PPV, and NPV, while the x-axis represents the four genetic subtypes (PTPN11, KRAS, NRAS, and NF1). Each subtype has four bars, corresponding to the four performance metrics. [file 13148_2025_1983_MOESM2_ESM.tif]

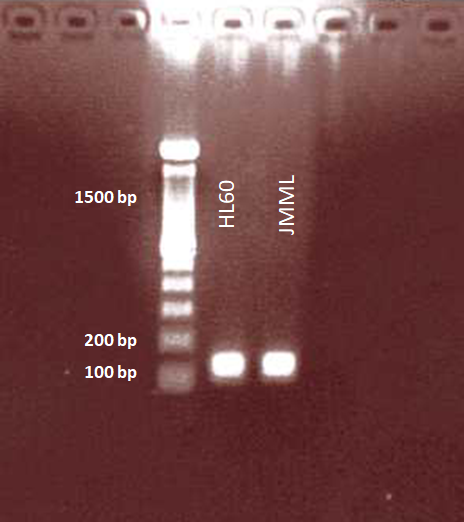

Supplement: Supplementary file 3 — Additional file 3. [file 13148_2025_1983_MOESM3_ESM.tif]
