# Supplementary material for: Epigenetic risk stratification in juvenile myelomonocytic leukemia by targeted methylation analysis of the BMP4 locus
Source: Clin Epigenetics. 2025 Oct 3;17:154. doi: 10.1186/s13148-025-01983-0 (PMC12492826; doi:10.1186/s13148-025-01983-0)
Supplement: Supplementary file 6 — Additional file 6. [file 13148_2025_1983_MOESM6_ESM.docx]

**Supplemental Table 3**

**A) Total cohort (N=111)**

|  | *BMP4*h | *BMP4*n |  |
| --- | --- | --- | --- |
| array HM | 19 | 12 | 31 |
| array non-HM | 9 | 71 | 80 |
|  | 28 | 83 | 111 |

**B) *PTPN11* cohort (N=40)**

|  | *BMP4*h | *BMP4*n |  |
| --- | --- | --- | --- |
| array HM | 14 | 7 | 21 |
| array non-HM | 3 | 16 | 19 |
|  | 17 | 23 | 40 |

**C) *KRAS* cohort (N=18)**

|  | *BMP4*h | *BMP4*n |  |
| --- | --- | --- | --- |
| array HM | 1 | 0 | 1 |
| array non-HM | 0 | 17 | 17 |
|  | 1 | 17 | 18 |

**D) *NRAS* cohort (N=19)**

|  | *BMP4*h | *BMP4*n |  |
| --- | --- | --- | --- |
| array HM | 3 | 2 | 5 |
| array non-HM | 2 | 12 | 14 |
|  | 5 | 14 | 19 |

**E) *NF1* cohort (N=13)**

|  | *BMP4*h | *BMP4*n |  |
| --- | --- | --- | --- |
| array HM | 1 | 3 | 4 |
| array non-HM | 2 | 7 | 9 |
|  | 3 | 10 | 13 |

**Supplemental Table 3.** Comparison of methylation categories as determined by *BMP4* bs-NGS or methylome-wide microarray.
